# A Lifestyle-Based Fuzzy-Enhanced ANN Model for Early Prediction of Type 2 Diabetes and Personalized Management in the North Indian Population

**DOI:** 10.3390/diagnostics15243139

**Published:** 2025-12-10

**Authors:** Shahid Mohammad Ganie, Majid Bashir Malik

**Affiliations:** 1 Department of Health Information Management and Technology, College of Applied Medical Sciences, King Faisal University, Al-Ahsa 31982, Saudi Arabia; 2 Department of Computer Sciences, Baba Ghulam Shah Badshah University, Rajouri 185234, Jammu and Kashmir, India

**Keywords:** type-2 diabetes mellitus, lifestyle indicators, self-management, predictive modeling, recommender system, ANN model, fuzzy membership function, knowledge-based system

## Abstract

**Background:** Type 2 Diabetes Mellitus (T2DM) continues to rise rapidly in Indian communities, affecting millions and posing a major public health challenge. Early identification of risk and timely lifestyle intervention are crucial for prevention. This study aims to develop a lifestyle-driven, fuzzy-enhanced Artificial Neural Network (ANN) model for early T2DM prediction and to design a personalized recommendation framework tailored to the North Indian population. **Methods:** A comprehensive exploratory data analysis, including statistical significance testing and age-cohort assessment, was conducted to evaluate data quality and identify key lifestyle associations. The ANN model was trained on 1939 lifestyle profiles and classified individuals into four risk categories: low, moderate, high-risk, and diabetic. A monotonic spline-based calibration method was used to refine predicted probabilities. Additionally, a web-based system, the Personalized Care and Intelligence System for Early Diabetes Assessment (PCISEDA), was developed to deliver individualized diet and physical activity recommendations. Cost-effective lifestyle options were curated via a structured web-scraping pipeline. **Results:** The proposed fuzzy-enhanced ANN model achieved an accuracy of 93.64%, precision of 94.00%, recall of 93.50%, F1-score of 93.50%, and a multiclass ROC–AUC of 94.07%, demonstrating strong discriminative performance. Feature importance analysis revealed age, weight, urination frequency, and thirst as the most influential lifestyle predictors of T2DM risk. The PCISEDA system successfully generated personalized and economically feasible lifestyle recommendations for each risk category. **Conclusions:** This lifestyle-based AI framework demonstrates substantial potential for early T2DM risk stratification and tailored lifestyle management. The integration of fuzzy calibration and personalized recommendations offers an accurate, scalable, and cost-effective solution that may support diabetes prevention and management in resource-constrained healthcare settings.

## 1. Introduction

Diabetes mellitus is considered one of the main healthcare issues that is affecting millions of people globally (https://www.who.int/health-topics/diabetes#tab=tab_1, accessed on 10 September 2025). It is a fatal disease and is caused by metabolic disorders in humans when the body cannot produce enough insulin or cannot effectively use the hormone properly [1,2]. Individuals with diabetes are trapped in the harmful deterioration of pancreatic beta cells due to a targeted autoimmune attack specifically focused on these cells [3]. It is a long-term and serious condition that reduces life expectancy and degrades the living standards of human beings. The mismanagement of diabetes can lead to severe complications by affecting several organs and tissues of the human body (https://my.clevelandclinic.org/health/diseases/7104-diabetes-mellitus-an-overview, accessed on 10 September 2025). Typical initial signs observed in diabetic and potentially diabetic patients include heightened thirst, extreme fatigue, weight fluctuations, dizziness, changes in skin color, sexual dysfunction, fungal infections, elevated blood sugar levels, and frequent urination [4]. Nearly 40 different types of diabetes exist, and most people worldwide are not aware of the complications associated with this disease due to inadequacies in healthcare resources and systems (https://diabetesatlas.org/resources/idf-diabetes-atlas-2025/, accessed on 12 September 2025). Some of the common types of diabetes are Type 1 Diabetes Mellitus, also called insulin-dependent diabetes, which is caused by autoimmune dysfunction and may occur at any stage, but is mostly observed in children and adolescents [5]. Type 2 Diabetes Mellitus, also called insulin-independent diabetes, is the most common type and occurs when the body does not produce or use insulin appropriately [4]. This type of diabetes can be controlled and managed by a healthy lifestyle. Another type is gestational diabetes, also called Type 3 Diabetes, which occurs in women during pregnancy because of hyperglycemia conditions and can even affect the baby’s health [6]. Finally, Type 4 Diabetes, also called prediabetes, is caused due to an increase in the production of insulin [7].

**Figure 1:**
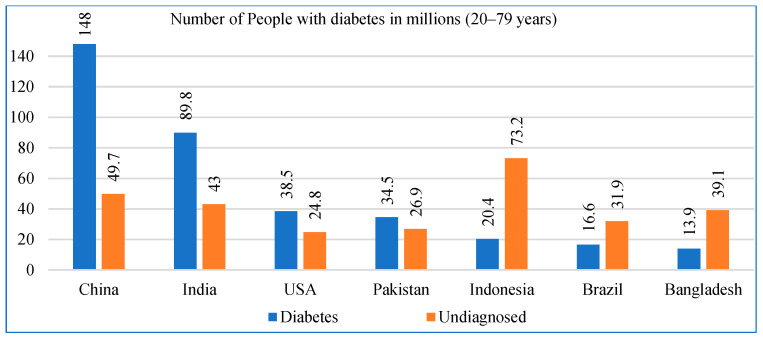
Top seven countries suffering from diabetes mellitus according to the IDF Atlas 2025.

According to the latest International Diabetes Federation (IDF) Diabetes Atlas 2025, approximately 560 million adults are living with diabetes globally, and the number is projected to reach 632 million by 2035 [8]. Figure 1 presents the estimates of diabetes prevalence and undiagnosed cases (ages 20–79 years) across the top seven countries most affected by diabetes, based on the IDF Diabetes Atlas 2025. China reports the highest number of adults living with diabetes (148 million), followed by India (89.8 million), the United States (38.5 million), Pakistan (34.5 million), Indonesia (20.4 million), Brazil (16.6 million), and Bangladesh (13.9 million). It also highlights the substantial burden of undiagnosed diabetes, which remains critically high in several countries, most notably Indonesia (73.2%), China (49.7%), India (43%), and Pakistan (26.9%). This visualization provides an updated overview of global diabetes distribution using the latest IDF 2025 modeling data.

Traditional clinical methods for diabetes detection, including pathways, reports, and tests, lack the ability to capture the dynamic and personalized nature of Type 2 Diabetes prediction [9,10]. These approaches ignore lifestyle parameters in disease prediction. The following techniques enable feature selection, risk assessment, predictive modeling, early detection, and personalized medicine. Fuzzification transforms precise data into fuzzy representation, incorporating uncertainty inherent in real-world data [11,12]. Integrating a predictive and personalized recommendation system into clinical workflows can improve self-management and early intervention [13]. Seamless connectivity with electronic health records (EHRs) enables automatic retrieval of demographics, medical history, and recent laboratory results, allowing clinicians to review AI-generated recommendations during consultations. Automated digital surveys supported by mobile technologies can routinely collect lifestyle data such as diet, physical activity, and symptom patterns, ensuring continuous risk monitoring [14]. Clinical decision support alerts may notify providers when a patient’s risk exceeds a threshold or when adherence declines, prompting timely follow-up. Such a system can also integrate with scheduling platforms, pharmacy systems, and telemedicine services, enabling automated appointment reminders, medication adherence alerts, and remote consultations, particularly valuable for rural populations with limited access to care. These features align with patient-centered care principles by ensuring timely support, personalized communication, and improved accessibility [15].

Despite significant progress in diagnostic technologies, conventional screening approaches often fail to capture early-stage risk, especially among individuals who exhibit mild or lifestyle-driven symptoms [16]. Early prediction using lifestyle indicators, rather than solely clinical biomarkers, offers a promising, low-cost strategy for identifying at-risk individuals and delaying disease progression [17,18]. With the increasing adoption of AI-driven predictive analytics, there is a growing opportunity to integrate machine learning with personalized lifestyle recommendations to support self-management and preventive care [19,20].

The primary objective of this research is to develop an integrated, lifestyle-centric Artificial Intelligence framework for realistic and early-stage healthcare management of Type 2 Diabetes Mellitus (T2DM). The proposed system incorporates two major components: (i) an ANN-based predictive analytics module enhanced with fuzzy logic for multi-class risk stratification, and (ii) a personalized recommender system that generates diet and physical activity suggestions tailored to each risk category. The key contributions of this work are summarized as follows:

- **Identification of lifestyle indicators:** We identified, refined, and collected lifestyle parameters (e.g., thirst, urination frequency, fatigue, smoking, drinking, anthropometrics, and family history) in consultation with endocrinologists, diabetologists, and nutritionists to ensure clinical relevance and real-world applicability.
- **Exploratory data analysis:** Extensive exploratory data analysis (EDA) was performed, including duplicate detection, missing value assessment, outlier analysis (IQR method), and statistical significance testing (Cramer’s V + chi-square). No missing or corrupted data were found, ensuring a high-quality dataset for model development.
- **Development of ANN–fuzzy model:** We developed a deep ANN architecture (three hidden layers) enhanced with a spline-based fuzzy membership function for calibrating outputs and subdividing non-diabetic classes into low-risk (A), moderate-risk (B), and high-risk (C) groups. This hybrid ANN–fuzzy model provides interpretable and robust multi-class prediction for T2DM.
- **Personalized recommender system:** A rule-driven and cost-aware recommender system was designed to generate individualized diet plans and physical activity charts based on risk category, symptom intensity, and expert dietary guidance. Web-scraped nutritional and pricing data were incorporated to ensure affordability.
- **Knowledge-based interface:** A web-based interface was built to deliver predictions, risk explanations, and personalized recommendations to end users. This interface supports early monitoring, promotes self-management, and enhances doctor–patient engagement.

The remainder of this paper is structured as follows. Section 1 introduces Type 2 Diabetes Mellitus (T2DM), its clinical complications, and its global burden, supported by recent statistical evidence. Section 2 provides a comprehensive review of existing research on diabetes prediction models and personalized recommender systems and synthesizes the key research gaps motivating this study. Section 3 details the proposed methodology, including data acquisition, exploratory data analysis, correlation analysis, preprocessing, feature significance assessment, ANN-based multi-class prediction, fuzzy logic calibration, and training and testing configurations of the proposed model. Section 4 presents and discusses experimental findings, including feature importance, model performance, comparative evaluation with existing models, and class-wise recommendations. Finally, Section 5 concludes the study by summarizing key contributions, outlining the limitations, and suggesting future research directions to enhance scalability, clinical integration, and real-time applicability.

## 2. Literature Review

AI-based approaches with different computational models have contributed significantly to the field of healthcare and have the potential to assist in early detection, intervention, and effective management of multiple chronic diseases, like diabetes, heart disease [21], obesity [17], cancer, etc. [22]. A healthy lifestyle is a key indicator of these chronic diseases, especially diabetes. In recent years, researchers and healthcare professionals worldwide have employed various computational models to predict the onset of Type 2 Diabetes. Several approaches that have been implemented are as follows:

Kumar et al. [21] developed a framework to predict diabetes based on deep neural network using the publicly available PIMA dataset. The authors implemented the model using various statistical measures such as accuracy, specificity, sensitivity, and recall. The model accomplished 98.16% accuracy using a random train–test split, and the proposed model acquired better results than state-of-the-art methods. Rahman et al. [22] developed a novel model for the classification of diabetes. The authors utilized a combination of a convolutional neural network (CNN), traditional long short-term memory (LSTM), and CNN-LSTM models, and then compared their performance with the developed model using the PIMA Indians Diabetes Database (PIDD). The Boruta algorithm was employed to extract significant features. Kannadasan K et al. [23] presented a model for diabetes classification based on deep neural network, employing stacked autoencoders that improved accuracy and enhanced other evaluation metrics. The parameter tuning of the DNN framework has been performed in a supervised fashion. Features were extracted from the dataset using stacked autoencoders and the dataset was classified via a SoftMax layer. To validate results, various evaluation metrics like precision, recall, specificity, and F1-score were calculated. The DNN achieved an accuracy rate of 86.26%, which outperformed various existing classification methods. Dahiwade et al. [24] designed a model based on k-nearest neighbor and a convolutional neural network using the PIMA diabetes dataset sourced from the UCI machine learning repository. The authors developed a framework to identify risk factors in patients based on a binary classification approach. For the sickness forecast, this research utilized KNN and CNN classifiers for an exact forecast of diabetes risk factors. CNN achieved the highest accuracy rate of 84.5%. In addition, as far as time and space complexity are concerned, KNN requires more resources than CNN. Huang and Lu [25] proposed a model that combines information gain and deep neural networks to reduce the complications and severity of diabetes. The authors used the Waikato Environment for Knowledge Analysis (WEKA) tool for implementation and to select optimal attributes in order to identify the risk factors towards diabetes. The developed model achieved an accuracy rate of 90.26%. Joshi and Borse [26] developed a GUI tool by using the R2015a (MATLAB version 8.5) integrated development environment to predict whether a patient is diabetic. The backpropagation neural network was trained and tested to build software that acts as a medium between the doctor and patients. The dataset used contained eight contributing parameters to identify diabetes. As far as results are concerned, BPNN achieved an accuracy rate of 81%, with a smaller number of iterations and a minimum Mean Square Error (MSE) rate of 0.107%. Sumi and Balachandran [27] developed a predictive model using various multilayer perceptron (MLP) algorithms for diabetes. The experimental study utilized the Matlab R2013 integrated development environment and the PIMA diabetes dataset. The results revealed that among all the algorithms, the Levenberg–Marquardt algorithm demonstrated optimal performance in terms of various matrices, like precision, recall, and F1-score.

Personalized recommender systems for diabetes management can be developed based on various sources, including clinical pathways, hospital repositories, laboratory tests, electronic health records (EHRs), and lifestyle indicators from multiple applications. By analyzing each source, recommender systems can offer tailored suggestions for medication regimens, diet plans, exercise routines, and lifestyle modifications. These systems leverage AI-based techniques to adapt recommendations based on individual preferences, clinical parameters, and responses to interventions. Here are some main approaches to building such systems: Nguyen et al. [28] presented a case study focusing on the correlation between lifestyle, diet, and the risk of developing Type 2 Diabetes Mellitus in Vietnam. Data from 1100 patients aged 40 to 65 years were collected for the research. In this work, the authors recognized the significance of lifestyle and dietary patterns among the Vietnamese population. Different statistical observations were made from the data samples to examine the relationship between diet and lifestyle with respect to T2DM. Balducci et al. [29] performed a systematic review related to the effect of diet on Type 2 Diabetes Mellitus. They identified and demonstrated various diet charts related to improvements in metabolic conditions. Patients with Type 2 Diabetes can look over numerous useful dietary regimens according to their preferences and social customs. The authors suggested that weight is a significant risk factor for different chronic diseases. Alian et al. [30] proposed a proactive personalized recommender system specifically for American Indians (AI) to fight against diabetes. A mobile application was used as a platform to gather information for smart healthcare systems for AI patients. Evaluation of the system was based on use cases and human expert verification, demonstrating the reliability and validity of the system. The authors proposed that this research can be extended to different people all over the globe by collecting patient information and using the same recommended system for diabetes prevention. Aklima et al. [31] developed a framework for a diet model among patients suffering from Type 2 Diabetes in Indonesia. For observation, sixty patients suffering from T2DM were selected from four villages of the Banda Raya Community Health Centre in Banda Aceh, Indonesia. The results showed a moderate level of dietary behaviors among patients with T2DM, but the identification of required caloric intake was at a low level. So, the authors suggested that this work can be extended to develop a realistic framework for dietary self-management using an intervention approach to improve the diet of patients suffering from Type 2 Diabetes Mellitus. Karami et al. [32] implemented a framework by analyzing public opinions related to diabetes, including diet, exercise, and obesity (DDEO), extracted from tweets. Analysis of a dataset comprising 4.5 million tweets showed that 8% of the tweets discussed diabetes, 23.7% mentioned diet, 16.6% referenced exercise, and 51.7% addressed obesity. The research revealed that the most significant correlation was between exercise and obesity, followed by diabetes and obesity. These data can serve as valuable support for clinical providers and public health experts to gain better insights into prevailing public opinions regarding diabetes, diet, exercise, and obesity. Mohammed and Hagras [33] designed a diet recommendation system based on fuzzy logic for diabetes. They provide a balanced diet plan for patients to achieve a healthy lifestyle and to control and reduce complications of the disease. The authors suggested that the proposed white-box (T2FS) model can be used for large datasets, along with the consultation of domain expertise, to achieve better results. Sharawat and Dubey [34] developed a diet model for diabetes using an analytical hierarchy process (AHP) based on multi-criteria decision-making (MCDM) method. The proposed model presented an optimum collection of various types of food based on various benchmarks. The authors suggested that this diet can be used in daily life to help prevent diabetes, leading to a healthy infancy, which is very important for a country and contributes to hale and hearty surroundings.

However, some limitations are evident across the existing literature. First, most predictive models are trained on clinical or benchmark datasets (e.g., PIMA) and focus on binary diagnosis rather than early risk stratification based purely on lifestyle indicators, which are more accessible in low-resource settings. This restricts their applicability to individuals who present with clear symptoms or have undergone clinical testing, thereby missing the window for early, lightly symptomatic, or pre-symptomatic prediction. Second, many recommender systems provide generic or rule-based advice and are not tightly integrated with a data-driven risk prediction model; they rarely adapt recommendations dynamically to an individual’s evolving lifestyle profile, sociodemographic context, and risk level. Third, there is a scarcity of large, lifestyle-centric datasets and end-to-end frameworks that simultaneously offer (i) risk prediction, (ii) multi-level patient stratification, and (iii) personalized, yet cost-effective, recommendations validated by domain experts.

To address these gaps, we propose an integrated lifestyle-based AI framework for T2DM that combines an ANN-driven risk prediction model, fuzzy logic-based risk stratification, and a personalized recommender system for diet and physical activity. Unlike prior work that relies predominantly on clinical variables, our model is trained on a real-world lifestyle dataset covering a broad age range (5–83 years) and uses routinely observable factors such as age, weight, thirst, urination, fatigue, smoking, drinking, and family history. The ANN component performs multi-class risk prediction, the fuzzy inference engine refines risk categories using expert-defined linguistic rules, and the recommender module generates individualized, economically feasible lifestyle plans tailored to each risk stratum. By focusing on early prediction from lifestyle data and tightly linking prediction with personalized recommendations, the proposed framework aims to operationalize scalable, low-cost, and patient-centered T2DM prevention and management in everyday settings.

## 3. Methodology Adopted

**Figure 2:**
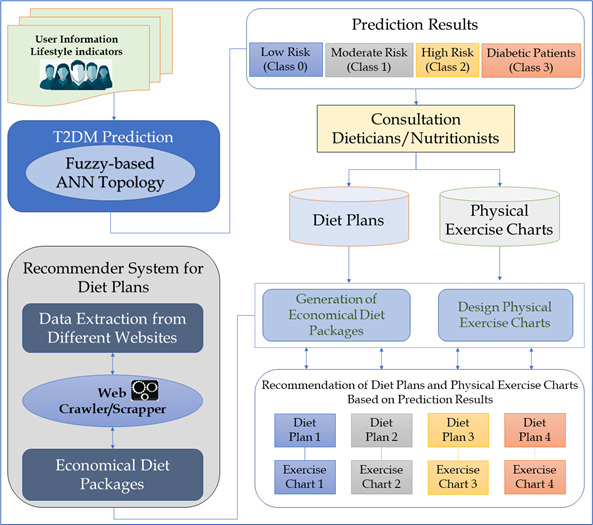
Proposed methodology of the research work.

Figure 2 illustrates the overall workflow of the proposed lifestyle-based fuzzy-enhanced ANN framework for early prediction and personalized management of Type 2 Diabetes Mellitus (T2DM). The methodology begins with the collection of user-specific lifestyle indicators, which serve as input to the ANN-based prediction module. The ANN model classifies individuals into four categories, including low-risk, moderate-risk, high-risk, and diabetic, based on their lifestyle patterns. These prediction results are then integrated with expert-curated knowledge from dieticians and nutritionists to generate personalized recommendations. Also, recommender system processes the predicted risk category and provides tailored diet and physical activity plans. This system incorporates a web-scraping module that extracts cost-effective dietary options from multiple online sources, enabling the construction of affordable meal packages. In parallel, expert-designed physical exercise charts are mapped to each risk class. The final output consists of individualized diet plans and exercise charts aligned with the user’s risk level, supporting early intervention, lifestyle modification, and continuous self-management.

**Figure 3:**
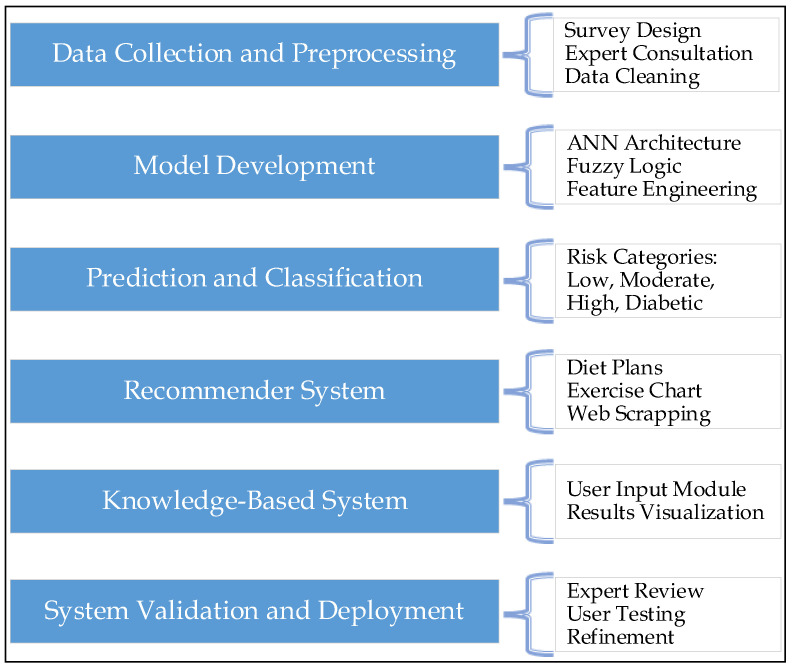
Comprehensive framework (PCISEDA) for T2DM prediction and personalized management system.

Figure 3 illustrates the end-to-end development process of an AI-driven system for Type 2 Diabetes Mellitus (T2DM) prediction and management. The focus is on the convergence of AI techniques and domain expertise in a user-centric, modern platform that brings together clinical insights and effective lifestyle intervention. The Personalized Care and Intelligence System for Early Diabetes Assessment (PCISEDA) system incorporates several components, such as data collection from various sources, preprocessing methods, data prediction through an ANN model, a classification system based on fuzzy logic, and a recommender system for personalized lifestyle modification recommendations. Overall, PCISEDA can be viewed as a comprehensive approach to preventing and controlling diabetes, as it focuses on early detection and lifestyle changes to prevent progression. In this way, this system not only assists medical professionals with data processing and decision-making, but it also enables people to make informed decisions to prevent T2DM in their lives or at least delay its onset.

### 3.1. Data Selection and Collection

**Figure 4:**
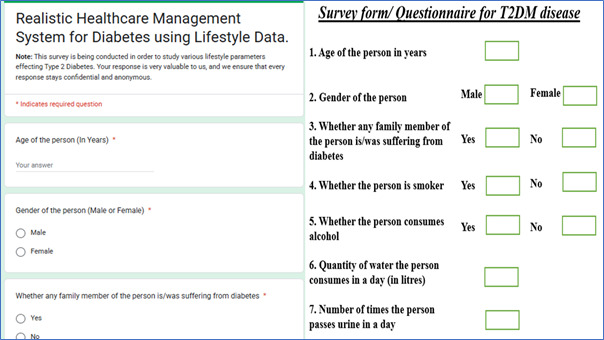
Google Forms and survey form/questionnaire for data collection.

Data is collected through survey forms/questionnaires and Google Forms, as shown in Figure 4. They were designed after selecting lifestyle indicators in consultation with domain experts, such as diabetologists and endocrinologists. Data from hospitals were collected through survey forms, while Google Forms were shared among diverse groups of individuals, ensuring a good combination of patient/candidate participants, like people from different areas, male–female ratio, patients from various classes (urban and rural areas), and adults from different age groups. The selected parameters/attributes were intentionally kept simple, as they are commonly known and represent outcomes of sedentary lifestyles and poor dietary habits. Importantly, no prior knowledge or awareness about the disease was necessary, and clinical advice or guidance was not required for participants to provide the requested data.

### 3.2. Parameter Information

The dataset contains 1939 records with 11 biological and lifestyle parameters. The first ten parameters are predicators, and the last one is the target variable. Table 1 presents the attribute descriptions, measurement units, and the range of values for each parameter.

### 3.3. Exploratory Data Analysis

Exploratory data analysis (EDA) is a crucial technique used to uncover meaningful patterns and insights within a dataset, essential for various tasks [35]. It involves the application of descriptive statistics to data, helping us gain a thorough understanding of its characteristics and structure. EDA for data preprocessing includes data wrangling, data standardization, and data transformation to improve the quality assessment of the considered dataset. In the following subsections, we present and discuss the obtained results.

#### 3.3.1. Descriptive Statistics of Parameters

Table 2 displays statistical measurements for the dataset’s lifestyle parameters. These include record count, min, 25%, 50%, and 75% frequencies, max, mean, and Std for each parameter. For instance, age has 1939 records, with mean of 41.77 and a Std of 15.84, ranging from 5 to 83. The same statistical analysis applies to other parameters present in the considered dataset.

#### 3.3.2. Age-Wise Distribution

The age-cohort analysis examines the distribution of participants across the five age groups (5–17, 18–30, 31–45, 46–60, >60 years) and the corresponding prevalence of Type 2 Diabetes. As presented in Table 3, diabetes prevalence increases steadily with age, rising from 2.7% in the youngest cohort to 47.5% among individuals above 60 years. While the majority of participants fall within the 31–45 age group, the highest disease burden is concentrated among older adults. This pattern highlights a strong age-related trend in diabetes risk, confirming that susceptibility increases significantly after age 45. The high prevalence in older cohorts emphasizes the need for targeted screening and preventive strategies.

Although the dataset spans a wide age range (5 to 83 years), we employed a unified ANN-based model for prediction due to the absence of sufficient cohort-specific training samples, especially in younger age brackets. Preliminary stratified performance checks across age cohorts (children < 18, adults 18–60, seniors > 60) revealed that the unified model retained high classification accuracy (>90%) across all cohorts without notable loss in sensitivity or specificity. Incorporating age as an input lifestyle indicator allowed the model to implicitly adjust predictions based on age-specific patterns. The findings support the modeling approach used in this study by demonstrating that age is a critical predictor for early diabetes risk assessment and must be incorporated into lifestyle-based AI frameworks for accurate early detection.

#### 3.3.3. Data Preprocessing

Data preprocessing is a crucial step in ensuring the integrity and reliability of the model-building process [36]. In this study, we applied the isnull() function and the SimpleImputer strategy to inspect the dataset for missing values. We also executed additional validation steps, including duplicate detection, corruption checks, and parameter-level consistency verification. No missing values, duplicate entries, or corrupted records were identified during this assessment, indicating that the dataset was already clean and complete. To investigate the presence of outliers, we used the Interquartile Range (IQR) method; however, no significant outliers were detected across the lifestyle variables.

**Figure 5:**
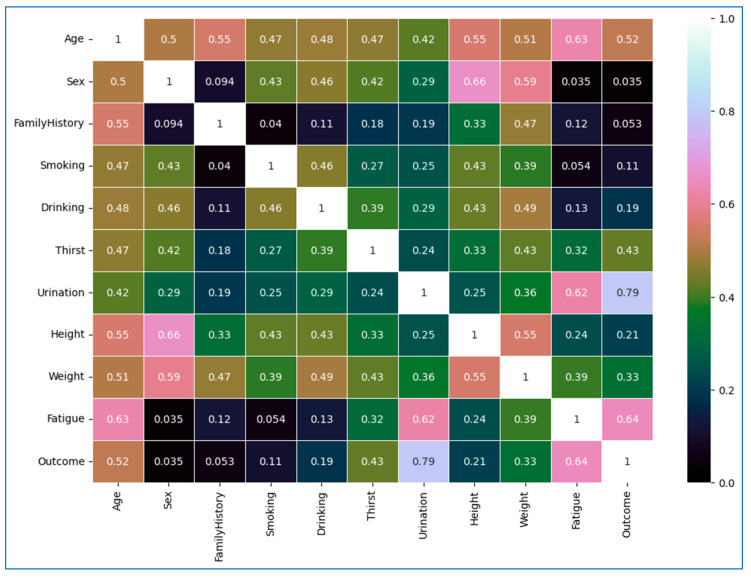
Cramer’s V association matrix, which summarizes effect sizes between lifestyle variables. Pairwise statistical significance for all variable combinations was additionally computed using chi-square tests. Only associations with *p* < 0.05 were treated as statistically significant during interpretation, although individual *p*-values are not embedded in the heatmap to maintain visual clarity.

To assess the statistical strength of associations between the categorical lifestyle variables and the diabetes outcome, we used Cramér’s V correlation matrix, as presented in Figure 5. To further strengthen interpretation, the statistical significance of each association was evaluated using the chi-square test of independence. All correlation values accompanied by *p* < 0.05 were considered statistically significant. The analysis confirmed that several lifestyle factors, particularly age, urination frequency, thirst, fatigue, weight, and family history, show statistically significant associations with diabetes outcome (*p* < 0.05), indicating that these variables meaningfully contribute to risk differentiation. Conversely, variables with lower Cramér’s V values and non-significant *p*-values (*p* ≥ 0.05), such as sex and smoking, demonstrated weaker or negligible relationships with diabetes status. Incorporating statistical significance supports the robustness of the correlation matrix and validates the inclusion of the most influential lifestyle predictors in the subsequent modeling process.

Table 4 presents the association between each lifestyle factor and the presence of Type 2 Diabetes (T2DM), quantified by an appropriate correlation coefficient (Cramer’s V) alongside the *p*-value from a chi-square test of independence. Because Cramer’s V does not provide directionality, the indicated arrows reflect the empirical trend observed in the data (e.g., higher weight more common in diabetics), rather than positive or negative correlation in the statistical sense. Notably, *age*, *weight*, *frequency of thirst*, *frequent urination*, *self-reported fatigue*, and *family history* of diabetes all show moderate correlation coefficients and reach statistical significance (*p* < 0.05). These significant associations suggest that individuals who are older or have higher body weight, experience excessive thirst and urination, report chronic fatigue, or have a family history of diabetes are more likely to have T2DM. In contrast, factors *such as sex*, *smoking status*, *alcohol drinking habits*, and *height* exhibit low correlation values that do not attain significance (*p* ≥ 0.05). This indicates that gender and these particular lifestyle choices or traits have only a weak or negligible relationship with diabetes in our dataset.

The inclusion of *p*-values and significance indicators underscores the robustness of the observed correlations and directly addresses the need for statistical support in our analysis. Associations accompanied by *p* < 0.05 validate that the correlations are unlikely due to chance, reinforcing the importance of the corresponding lifestyle factors in relation to T2DM. For instance, the significant correlations for *age*, *weight*, *thirst*, *urination frequency*, *fatigue*, and *family history* confirm that these factors are non-randomly linked to diabetes outcomes, lending credibility to their role as key predictors. Conversely, the lack of statistical significance for sex, smoking, drinking, and height aligns with their low correlation values, confirming that any observed relationship for these features is not statistically meaningful.

### 3.4. Diet Plans and Physical Exercise Charts

Recent research studies in medical sciences have proven experimentally that diet and exercise play an important role in controlling, delaying, and managing diabetes, especially Type 2 Diabetes Mellitus [37,38,39]. Existing literature demonstrates relationships/associations between diet and exercise plans and various chronic diseases [40]. To mitigate the risk of developing diabetes in the future, it is essential to consistently monitor and maintain current lifestyle indicators, including factors such as height, weight, obesity, diet, food consumption, and exercise habits [41]. By regulating insulin levels in the blood, a well-balanced diet and regular physical exercise can assist in maintaining optimal blood sugar levels in both diabetic and potentially diabetic individuals [42].

### 3.5. Artificial Neural Network

**Figure 6:**
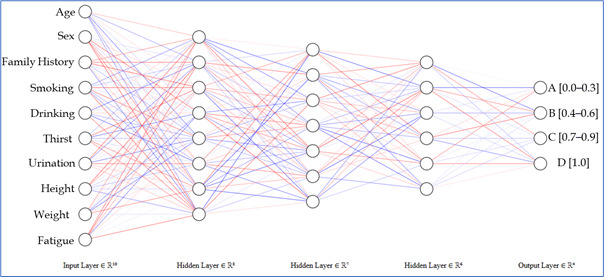
ANN topology for T2DM prediction.

An Artificial Neural Network (ANN) is a computational model inspired by the structure and functioning of biological neurons, capable of learning complex nonlinear relationships from data [43]. The network architecture used in this study consists of three hidden layers (as shown in Figure 6), enabling deep feature extraction and robust classification of lifestyle-based risk patterns related to Type 2 Diabetes Mellitus (T2DM). Equation (1) represents the computation within a single neuron (typically within an input or hidden layer), which is a component of the larger ANN topology.

(1) $$y_{i}=\emptyset \;(\;\overset{n}{\underset{i=1}{\sum }}w_{ij}x_{j}+b_{i})\;$$

where $y_{i}$ the predicted output for class i, ∅ is the activation function (ReLU for hidden layers, Softmax in the output layer), $x_{j}$ denotes the *j*-th input feature, $w_{ij}$ is the weight connecting feature j to output node i, and $b_{i}$ is the bias term.

### 3.6. ANN Architecture and Topology

We employed the ANN architecture for prediction of the considered disease, as shown in Figure 6. The input to the ANN consists of all predictor variables and input features. Artificial Neural Networks (ANNs) were well-suited for this research, as evident from previous studies. ANNs excel at handling complex, nonlinear relationships in datasets through their multi-layered structure, enabling deep feature extraction and meaningful output processing. ANNs are highly versatile and capable of learning, improving performance by adjusting weights using backpropagation algorithms to reduce errors. They can generalize from training data to unseen data, making them effective predictors. Their flexibility allows applications ranging from image recognition to disease diagnosis. Due to their capacity for integration and learning from diverse data sources, they represent an essential tool that can solve many problems with high accuracy. ANNs were used as they can approximate complex relationships, adapt, and learn better than classic methods, and have been successfully applied across various fields.

In this architecture, three hidden layers were used during experimentation and tuning. A spline-based membership function divides the non-diabetic class to identify the probability of future disease involvement. Non-diabetic patient records are divided into the following sub-classes: low-risk (A), moderate-risk (B), and high-risk (C) using reverse feature engineering. The four classes—A, B, C, and D—present the output. The results are discussed in the following subsections.

#### 3.6.1. Fuzzy Membership Function

**Figure 7:**
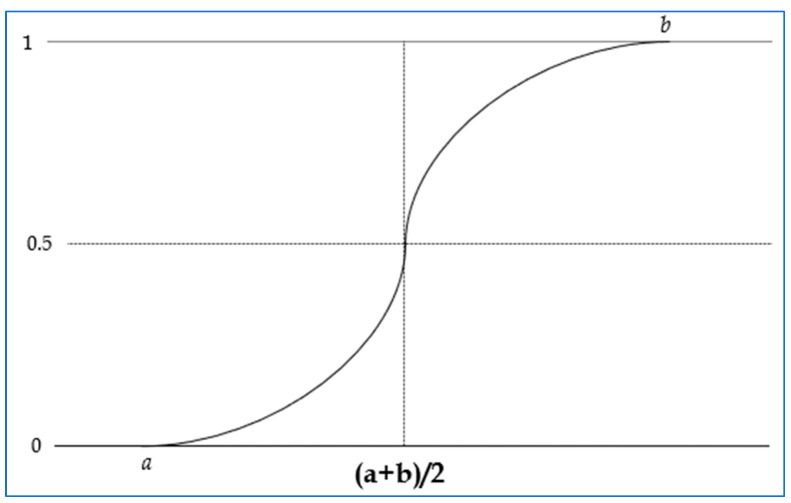
Curve of spline-shaped membership function.

Fuzzy logic is widely used in fields like artificial intelligence, where imprecision or vagueness exists in data values with respect to the outcome of a problem [44]. Fuzzy logic offers a straightforward approach to interference, even when dealing with noisy, vague, ambiguous, imprecise, or incomplete data. It operates as a multi-valued logic that approximately handles reasoning, prioritizing flexibility over precision [45]. In this work, the fuzzy S-shaped membership function (Equation (2)) is applied solely for deriving a continuous risk score that is subsequently used to subdivide the non-diabetic population into three meaningful risk groups (classes A–C). Only the derived risk score is fuzzified; the original lifestyle parameters remain unchanged and are not used in their fuzzified form for model training. Therefore, the fuzzified values represent the risk score transformation used for class labeling, whereas the ANN receives the normalized original feature values as input. This ensures that fuzzification influences only the generation of risk-based class labels, not the feature representations used by the ANN. The parameters ‘a’ and ‘b’ correspond to the endpoints of the inclined segment of the curve. A visual illustration of the spline-shaped membership function can be observed in Figure 7. A membership function is defined by three key aspects: Core, Support, and Boundary and it scales down the data samples between “0.0 to 1.0” with a corresponding degree of membership.

(2) $$\mu \left(x;a,b\right)=\left\{\begin{array}{c} 0,\;\;\;\;\;\;\;\;\;\;\;\;\;\;\;\;\;\;\;\;\;\;\;\;\;\;x\leq a\\ 2{\left(\frac{x-a}{b-a}\right)}^{2},\;\;\;\;\;\;\;\;\;\;\;\;\;\;\;\;\;\;\;\;\;\;\;\;a\leq x\leq \frac{a+b}{2}\\ 1-2{\left(\frac{x-b}{b-a}\right)}^{2},\;\;\;\;\;\;\;\;\;\;\;\;\;\;\;\;\frac{a+b}{2}\leq x\leq b\\ 1,\;\;\;\;\;\;\;\;\;\;\;\;\;\;\;\;\;\;\;\;\;\;\;\;\;\;x\geq b \end{array}\right\}\;$$

#### 3.6.2. Fuzzy Logic-Based Probability Calibration

The fuzzy logic-based spline calibration described in this section is applied exclusively to the ANN output probabilities, not to the raw lifestyle input features. After the ANN produces the predicted probability vector, a class-wise monotonic spline function is fitted to refine these probabilities. This calibration step adjusts the ANN outputs to improve probability reliability and enforce normalization across the four T2DM risk classes. No fuzzification or spline transformation is applied to input features at any stage; the only fuzzified values in the model pipeline are those used earlier for generating class labels (Section 3.6.1).

To improve the reliability of the ANN-predicted probability scores, we apply a monotonic spline-based calibration method inspired by isotonic regression. In this approach, the raw predicted probabilities for each class are first sorted and then fitted with a monotonic cubic regression spline that enforces non-decreasing behavior across the probability range. This ensures smooth, well-behaved calibrated scores while preserving the ranking of samples. The calibrated class-specific spline functions are subsequently normalized across all classes so that the resulting probability vector remains within the m-dimensional probability simplex. The overall calibration workflow is formally described in Algorithm 1.

Let

(3) $${\hat{P}}^{\left(k\right)}={\hat{P}}_{1}^{\left(k\right)},\;{\hat{P}}_{2}^{\left(k\right)},\;{\hat{P}}_{3}^{\left(k\right)},\;{\hat{P}}_{4}^{\left(k\right)}$$

be the ANN-predicted probability vector for sample k, where

(4) k=1,2,3,4…………..n 

and n=1939. For each class $i\in \left\{1,2,3,4\right\}$ we construct the corresponding one-vs-all indicator label:

(5) $$y_{i}\left(k\right)=\left\{\begin{array}{l} 1,\;if\;sample\;k\;\mathrm{belongs}\;\mathrm{to}\;\mathrm{class}\;i,\\ 0,\;otherwise. \end{array}\right.\;$$

A monotonic spline-based regression function $F_{i}\left(.\right)$ is fitted between the predicted probabilities and the true one-vs-all labels:

(6) $$F_{i}=SplineFit\;\left({\hat{P}}_{i}^{\left(k\right)}\;,\;y_{i}\left(k\right)\;\right)$$

After fitting the spline for each class, the calibrated probability for class i is computed as follows:

(7) $${\tilde{P}}_{i}^{\left(k\right)}=\;\frac{F_{i\;}{\hat{(P}}_{i}^{\left(k\right)})}{\sum_{j=1}^{4}F_{j\;}{\hat{(P}}_{j}^{\left(k\right)})}$$

This normalization guarantees:

(8) $$\sum_{j=1}^{4}{\tilde{P}}_{i}^{\left(k\right)}=1,\;\forall k\;\;:$$

**Algorithm 1:** Spline-Based Multi-class Calibration**Input**:
$\hat{P}$ 
∈ 
${\left[0,1\right]}^{n\times m}$: predicted probability matrix from the ANN.             
$Y\;\in {\left\{1,2,3,4,\ldots \ldots \ldots \ldots ..m\right\}}^{n}$: true class labels encoded as integers **Output**: A calibrated multiclass mapping
$F:\;\Delta^{m}\to \;\Delta^{m}$, where
$\Delta^{m}$ denotes the
m-dimensional probability simplex **1.**    For each class
k=1,2,…………..m:       **1.1**  Extract the raw predicted probabilities:         
${\hat{P}}^{k}=\hat{P}\left[:,k\right]$       **1.2**  Construct the one-vs-all indicator vector: for each sample
k=1,……….., n,         
$y_{i}\left(k\right)=\left\{\begin{array}{c} 1,\;\;if\;Y_{i}=k,\\ 0,\;\;otherwise. \end{array}\right.\;$       **1.3**  Sort probabilities for monotonic spline fitting:              •  Sort
${\hat{P}}^{k}$ in ascending order             •  Record
$y_{k}^{i}$ using the same sorted indices       **1.4**  Fit a monotonic cubic regression spline:              
$f_{k}=SplineFit\;{\hat{P}}^{k},\;y^{k}$              where SplineFit denotes a monotonic cubic spline fitted under isotonic (non-decreasing) constraints, ensuring smoothness and proper probability behaviour. **2.**    For
i=1,…………..n:            Compute the calibrated score for class
k:            
$F_{k}\left(x_{i}\right)=\;\frac{f_{k\;}\left({\hat{P}}_{i}^{k}\right)}{\sum_{j=1}^{m}f_{j\;}\left({\hat{P}}_{i}^{j}\right)}$         This normalization ensures that            
$\overset{m}{\underset{k=1}{\sum }}F_{k}\;\left(x_{i}\right)=1$ and that the final calibrated vector lies in the m-dimensional probability simplex
$\Delta^{m}$

#### 3.6.3. ANN Training and Testing Configuration

To ensure reproducibility and transparency, the complete training and testing configuration of the ANN model used for T2DM risk classification is summarized in Table 5. The dataset was split into 70% for training and 30% for testing, ensuring that all four classes (A–D) were proportionally represented in both sets. The ANN consists of three hidden layers and uses the ReLU activation function for all hidden layers, while the output layer employs Softmax for multi-class probability prediction. Model optimization was performed using the Adam optimizer, with categorical cross-entropy as the loss function. Training was conducted for 150 epochs with early stopping, a batch size of 32, and a learning rate of 0.001. This configuration allowed stable convergence, effective generalization, and robust classification performance across all four risk classes.

## 4. Experiment, Results, and Discussion

In this section, we present and discuss the outcomes of our proposed framework for a realistic healthcare management system for Type 2 Diabetes Mellitus. The results are directed towards improving self-care strategies for individuals affected by Type 2 Diabetes Mellitus (T2DM) through the utilization of lifestyle data.

### 4.1. Performance Evaluation of the Fuzzy-Based ANN Model

To extend binary classification to multi-class scenarios, we explored previous research works [46,47]. We applied the concept of smoothing spline function to be extended to higher dimensions, known as thin-plate splines. Nevertheless, directly extending this approach to the multi-class context becomes unfeasible due to the exponential increase in the required number of knots with the growing number of classes. To prepare the lifestyle dataset for predictive modeling, a structured feature engineering workflow was implemented, comprising normalization, fuzzy membership computation, and class-level risk categorization. All lifestyle parameters were normalized to the interval [0,1], as presented in Table 6, to ensure uniform scaling and prevent any feature from disproportionately influencing ANN training. This transformation preserves the underlying behavioral and physiological trends while improving numerical stability during model optimization.

Following normalization, a spline-based fuzzy membership function was applied to quantify the degree of association between an individual’s lifestyle indicators and their likelihood of developing Type 2 Diabetes Mellitus (T2DM). Unlike crisp threshold-based methods, the fuzzy membership function yields graded scores ranging from 0 to 1, capturing smooth and nonlinear transitions in symptom severity. This approach is particularly effective in early-stage or borderline cases, where risk patterns may not be distinctly separable through rigid clinical cut-off points. The resulting fuzzified values were subsequently mapped to four clinically interpretable T2DM risk classes:

- *Class A (0.0–0.3): Low-risk;*
- *Class B (0.4–0.6): Moderate-risk;*
- *Class C (0.7–0.9): High-risk;*
- *Class D (1.0): Confirmed diabetic.*

Table 6 presents the complete results of this feature engineering process, demonstrating how raw lifestyle attributes are systematically transformed into calibrated fuzzy scores and subsequently into risk categories. The table clearly shows the continuity between lifestyle patterns and disease likelihood, validating the fuzzy approach as an effective intermediary between raw data and predictive modeling. The outcomes of this feature engineering framework are crucial for both model performance and clinical interpretability. First, the fuzzy membership values enrich the ANN’s input structure by encoding nuanced symptom variations that may not be sufficiently captured by traditional normalization alone. Second, the risk categories (A–D) bridge the predictive ANN model and the personalized recommendation module, enabling the system to generate tailored diet and physical activity plans aligned with the user’s risk level. Finally, this methodology reinforces the model’s ability to detect early-stage T2DM risk by highlighting subtle but clinically relevant behavioral patterns, thereby enhancing both diagnostic precision and the practical utility of the recommender system.

**Table 6:** Results of feature engineering process.

S. No	Age	Urination	Thirst	Weight	Height	Fatigue	Outcome	Fuzzified Value	Class
1	0.340312	0.189349	0.09184	0.53635	0.245802	0.65691	0	0.100281	A [0.0–0.3]
2	0.617188	0.704142	0.17346	0.48773	0.539559	0.65691	0	0.749944	C [0.7–0.9]
3	0.572187	0.573964	0.16327	0.64762	0.371089	0.92228	0	0.570111	B [0.4–0.6]
4	0.772187	0.704142	0.2551	0.68785	0.723598	0.65691	1	1	D [1.0]
5	0.659688	0.573964	0.2551	0.60494	0.157675	0.92228	0	0.854766	C [0.7–0.9]
6	0.89875	0.704142	0.9898	0.37342	0.686438	0.65691	1	1	D [1.0]
7	0.327654	0.189349	0.09528	0.48773	0.09892	0.92228	0	0.000541	A [0.0–0.3]
8	0.834687	0.893491	0.97184	0.37342	0.136083	0.92228	1	1	D [1.0]
9	0.524687	0.704142	0.36735	0.46365	0.157675	0.65691	0	0.458965	B [0.4–0.6]
10	0.89875	0.704142	0.9898	0.72565	0.010796	0.92228	1	1	D [1.0]
11	0.360243	0.199675	0.09379	0.64765	0.245802	0.65691	0	0.100345	A [0.0–0.3]
.	.	.	.	.	.	.	.	.	.
.	.	.	.	.	.	.	.	.	.
1938	0.834687	0.893491	0.9898	0.37342	0.245802	0.92228	1	1	D [1.0]
1939	0.524687	0.704142	0.2551	0.46365	0.010796	0.92228	0	0.458965	B [0.4–0.6]

Note: The numerical lifestyle parameters presented in this table correspond to the normalized original dataset values. Only the final “Fuzzified Value” column reflects the S-shaped membership transformation used for class derivation. These fuzzified values are not used as ANN inputs; the ANN is trained exclusively on non-fuzzified lifestyle features, while the fuzzy spline calibration in Section 3.6.2 is applied only to ANN output probabilities.

**Figure 8:**
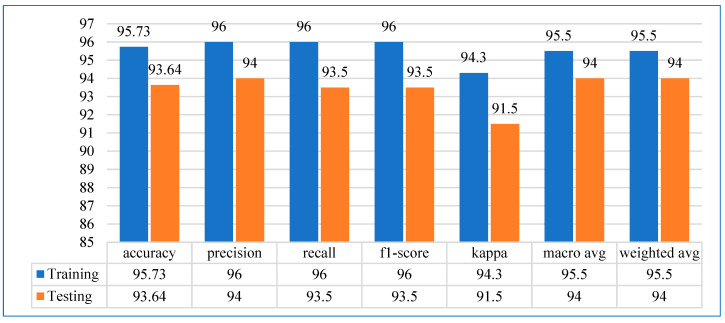
Performance evaluation metrics for training and testing datasets.

**Figure 9:**
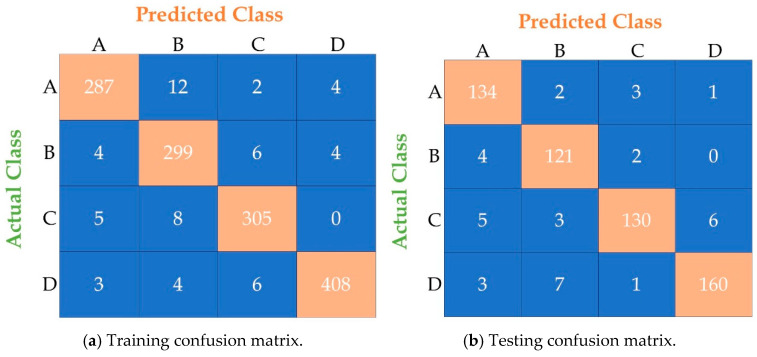
Confusion matrix for (**a**) training and (**b**) testing for the ANN Model.

Performance evaluation metrics assess a model’s efficacy, reliability, and robustness. These metrics provide insights into how well a model performs on a given task, whether classification, regression, clustering, or any other machine learning or statistical analysis. In our study, we evaluated the ANN model using various metrics, including accuracy, precision, recall, F1-score, kappa, macro avg, and weighted avg, on both the training and test sets. The ANN model achieved realistic results by metric analysis, as shown in Figure 8. The training and testing accuracies of the ANN model were 95.73% and 93.64%, respectively. In terms of precision, the model achieved 96% and 94% on the training and test sets, respectively. In addition, recall and F1-score for training and testing were 96% and 93.50%, respectively. The kappa scores for the ANN model’s training and test sets were 94.30% and 91.50%, respectively. The macro avg and weighted avg of the training and testing phases were 95.50% and 94%, respectively. The confusion matrix, presented in Figure 9, presents the relationship between the actual and predicted values.

**Figure 10:**
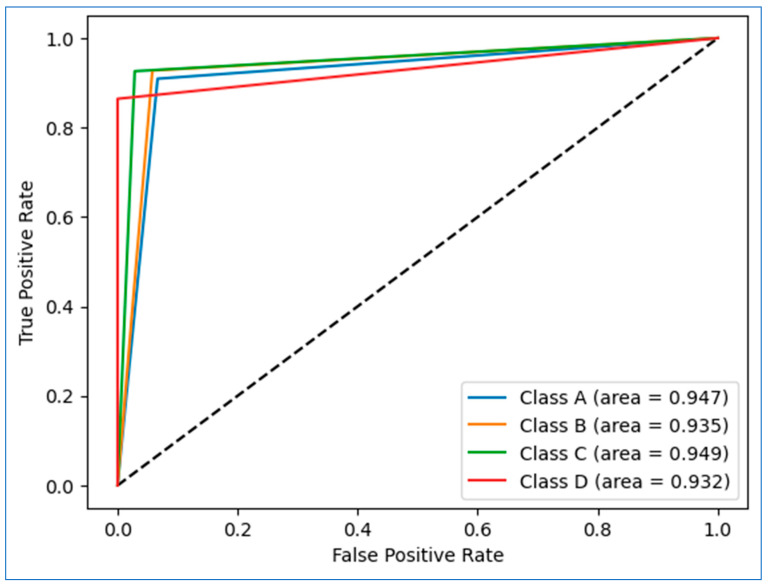
Each ROC curve represents true-positive rate vs. false-positive rate across all threshold values, and the AUC reflects the area under this curve, not just one threshold point.

Figure 10 presents the multi-class AUC-ROC curves for the ANN model across the four risk categories (Class A, Class B, Class C, and Class D). The ROC curves appear as piecewise-linear segments rather than smooth curves. This behavior arises because the ANN produces a limited number of distinct probability values for each class in the one-vs-rest evaluation setting, resulting in only a few threshold points for the computation of true-positive and false-positive rates. Despite the visual step-like form, the area under the curve (AUC) is computed over the complete set of threshold values and reliably reflects the discriminative performance of proposed model across all classes. The model demonstrates strong discriminative performance, with AUC values of 0.947 (Class A), 0.935 (Class B), 0.949 (Class C), and 0.932 (Class D). All curves lie well above the diagonal reference line, indicating that the classifier performs significantly better than random prediction across all classes.

Among the four categories, Class C shows the highest discriminative ability (AUC = 0.949), closely followed by Class A (AUC = 0.947), while Class D exhibits the lowest but still strong performance (AUC = 0.932). These consistent AUC values confirm stable and reliable risk stratification for each diabetes category. It can be observed that AUC values above 0.93 for all classes demonstrate excellent sensitivity–specificity trade-offs and suggest strong model generalizability across varying risk profiles, including low-risk (A), moderate-risk (B), high-risk (C), and diabetic (D) categories.

To quantify the discriminative strength of each lifestyle feature in separating the four T2DM risk classes (A–D), we compute feature importance using the ANOVA (Analysis of Variance) F-statistic. This method evaluates how much of the variance of a feature is explained by class differences relative to unexplained within-class variance. Given a feature *X* partitioned into *m* classes, the F-score is computed as follows:

(9) $$F=\frac{ss_{between}/\left(m-1\right)}{ss_{within}/\left(N-m\right)}\;$$

where

(10) $$ss_{between}=\overset{m}{\underset{i=1}{\sum }}n_{i}\;{\left(\mu_{i}-\mu \right)}^{2}$$

(11) $$ss_{within}=\overset{m}{\underset{i=1}{\sum }}\underset{k\epsilon i}{\sum }{\left(x_{k}-\mu_{i}\right)}^{2}$$

with $n_{i}$ as the number of samples in class *i*, $\mu_{i}$ as the mean of feature X in class *i*, μ as the overall mean across all samples, and N as the total number of samples.

**Figure 11:**
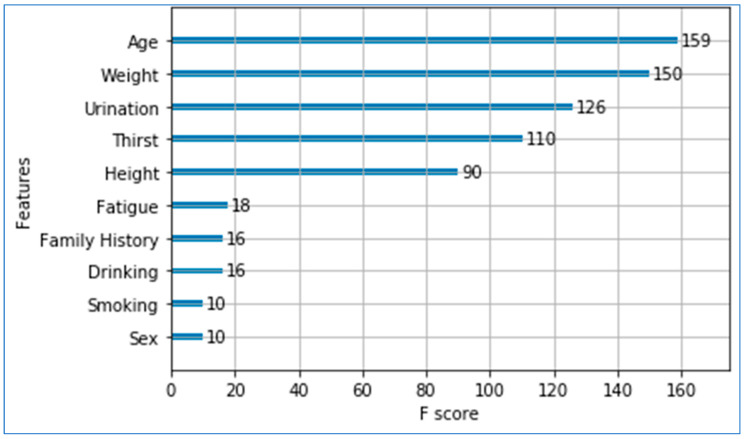
Feature importance scores for lifestyle predictors using the ANOVA F-statistic.

A higher F-value indicates that the feature exhibits stronger separation between classes, meaning its class-wise differences are significantly larger than the variability within each class. This makes the ANOVA F-statistic particularly suitable for ranking feature importance in multi-class prediction problems. Given that our features are continuous and normalized (range [0,1]), the ANOVA assumptions of normality, independence, and homogeneity of variance are reasonable and consistent with standard practice in feature selection. The resulting F-scores, presented in Figure 11, highlight the lifestyle factors most influential in predicting progression toward Type 2 Diabetes Mellitus. It can be observed that *age*, *weight*, *urination frequency*, and *thirst* are the four most influential lifestyle predictors, with F-scores of 159, 150, 126, and 110, respectively. *Height* also contributes moderately (F = 90), while features such as *fatigue*, family history, *drinking*, *smoking*, and *sex* show comparatively lower influence. These importance scores indicate the relative contribution of each variable toward the model’s decision-making process and highlight the lifestyle factors most strongly associated with T2DM risk in the analyzed population.

To evaluate the performance of the proposed ANN model, we conducted a comparative analysis against several state-of-the-art machine learning and ensemble methods reported in the literature. Table 7 summarizes the results across key evaluation metrics, including accuracy, precision, recall, F1-score, and AUC-ROC.

### 4.2. Recommender System for Economical Diet Plans and Physical Exercise Charts

**Figure 12:**
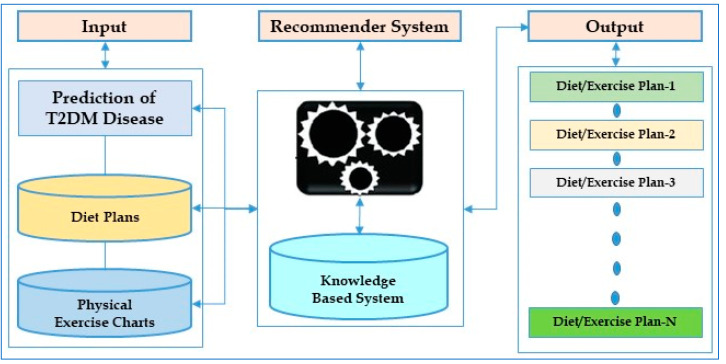
Recommender system for diet plans and physical exercise charts.

The proposed architecture, shown in Figure 12, outlines a workflow for recommending economical diet packages and physical exercise charts to potential and diabetic patients. In this framework, the results achieved through the ANN model using lifestyle parameters for prediction of T2DM are explored. These results were presented and discussed with experts, like dieticians/nutritionists, to design diet plans and physical exercise charts for different categories of patients. Also, a web crawler/web scraper was used to extract data regarding recommended diet plans in order to generate economical diet packages. Finally, the customized economical diet plans and physical exercise charts are recommended to all patient categories to follow healthy lifestyle habits in advance.

#### 4.2.1. Recommendation of Diet Plans

The growing economic burden of noncommunicable diseases like T2DM diabetes, cardiovascular diseases, and cancer, is increasingly recognized worldwide [53]. Hospital treatment and readmission costs are obstructions to adopting healthy eating habits, particularly individuals from lower socioeconomic backgrounds [54]. In addition, the cost of food items for patients having lifestyle-related diseases are a barrier to healthier diet plans. With the alarming rise of chronic degenerative diseases in many countries worldwide, prioritizing healthy eating habits can no longer be delayed [55]. Computational techniques can be used to develop an economical diet model that significantly reduces in healthcare costs associated with the management of T2DM by following dietary principles aligned with the Mediterranean diet. An economical diet model offers a platform to access and identify the most budget-friendly dietary items from various sources, tailored to different categories of individuals. The workflow for developing a framework for economical diet plans is discussed in the following subsections.

#### 4.2.2. Data Sources

**Figure 13:**
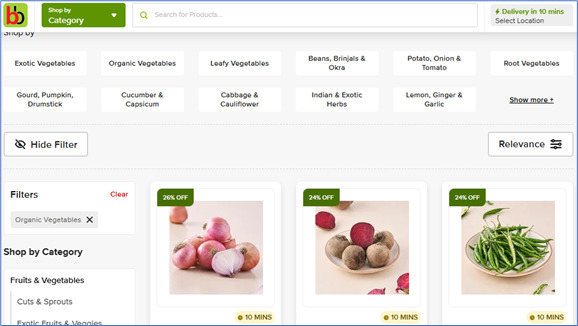
BigBasket website used to collect data for economical diet plans.

Various web sources were used to collect data on recommended diet plans. Popular online websites were being used as data sources, and an API (Application Program Interface) was developed to gather diet packages for T2DM patients. Websites like Bigbasket (https://www.bigbasket.com/), Sabzi Bazar (https://www.sabzibazar.co.in/), Nature’s Basket (https://www.naturesbasket.co.in/), and Flipkart (https://www.flipkart.com/) were used as data sites because these online grocery stores have a sufficient amount of daily diet items required by end users. Figure 13 depicts the website to collect the economical data showcasing BigBasket.

#### 4.2.3. Architecture for Development of Economical Diet Packages

**Figure 14:**
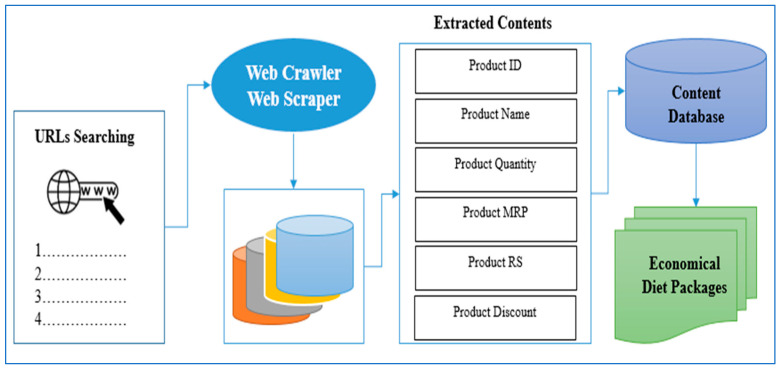
Architecture for economical diet packages.

**Figure 15:**
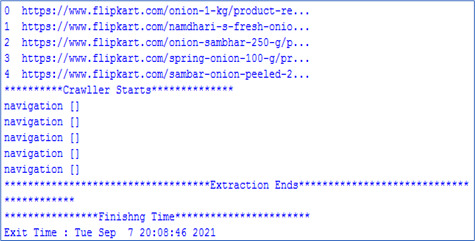
Output of execution for URLs searching.

**Figure 16:**
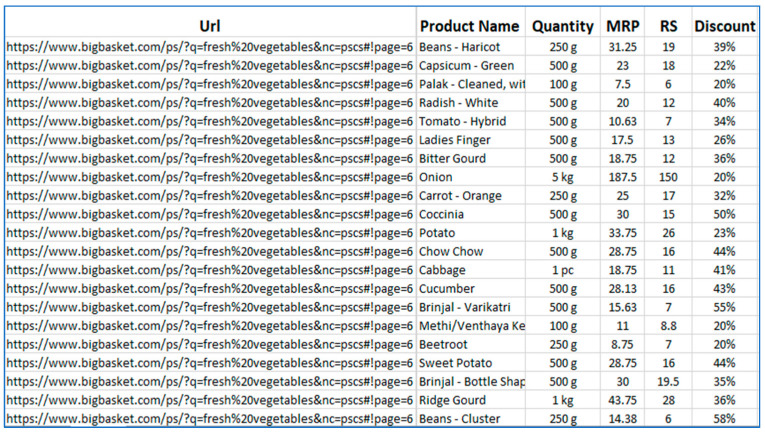
Extracted data related to diet plans from the Bigbasket website.

Web mining techniques were explored to extract data from different sources, which would be hard and inefficient to obtain manually [56]. In addition, manual extraction is time-consuming and not always free from errors and bugs. The architecture shown in Figure 14 illustrates the design of the economical diet packages. Data regarding diet plans from different online sites is accessed using the uniform resource locator of each webpage. Data regarding recommended diet plans is extracted using web scrapers like Data Miner (https://dataminer.io/), Webharvy (https://www.webharvy.com/), and import. IO (https://www.import.io/). The URLs are added to webcrawler/web scraper, which accesses the webpages and extracts the relevant information from the web content. An example of the scripting program execution for URL searching on the Flipkart website is shown in Figure 15. The extracted content is stored in a content database, as shown in Figure 16 for the Bigbasket website. The content database stores all the information scraped from webpages of different URLs. In addition, data from other websites has been extracted and stored in a content database. The data stored in content database were then compared in order to select the most economical diet packages for end users. Finally, these economical diet packages are designed to cater to different patient categories.

#### 4.2.4. Recommendation of Physical Exercise Charts

The physical exercise charts have been designed in consultation with domain experts for different categories of patients based on the prediction results. The prediction results were discussed in detail with experts and along with the contribution of each lifestyle parameter towards the disease. The dieticians and nutritionists provide a list of diet items and physical exercise charts for potentially diabetic and diabetic patients. The physical exercise charts for the different categories of patients are presented in Table 8 and Table 9. These customized physical exercise charts can help patients to follow healthy lifestyle habits in advance.

### 4.3. Knowledge-Based Interface

**Figure 17:**
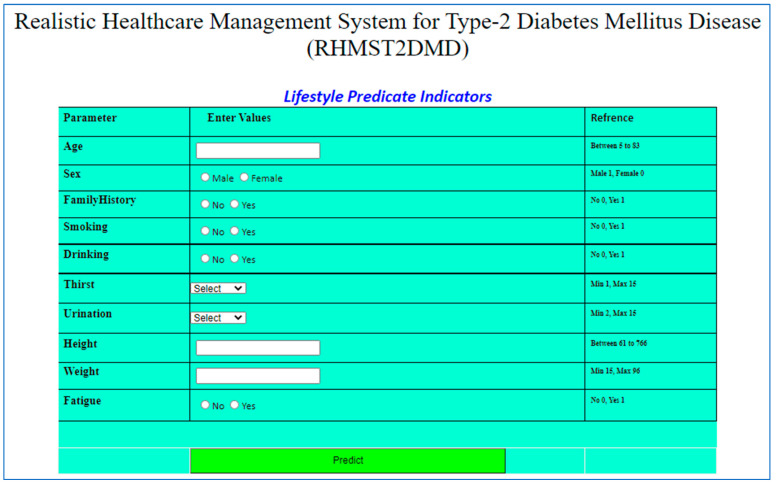
Graphical user interface for input of lifestyle parameters.

Artificial intelligence has rapidly emerged as the predominant technology in online systems, and designers can harness its capabilities to enhance web applications, enabling real-time predictions by inputting a few parameters and yielding superior results. A knowledge-based intelligent interface has been developed, i.e., Realistic Healthcare Management System Type 2 Diabetes Mellitus (RHMST2DM), for healthcare providers and end users. This web-based application is convenient, allowing users to easily provide input and receive their results. The web-based interface shown in Figure 17 is used to collect lifestyle data from users. An ANN developed and implemented a framework for prediction of T2DM is integrated with the backend of this interface to calculate the probability of disease.

**Figure 18:**
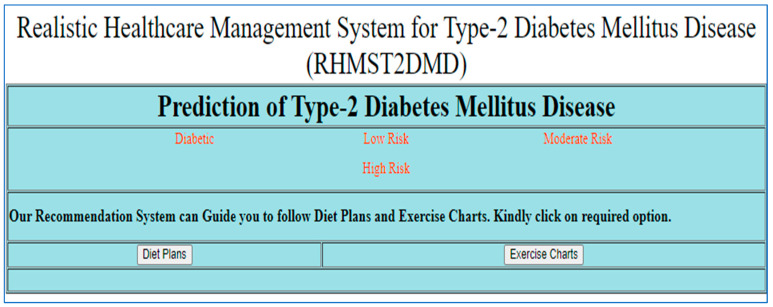
Classification of predicted results towards T2DM.

However, the recommendations are usually general and do not take into account the special properties of the potential patients with the disease [37]. Proper recommendations for diet plans and physical exercise charts are still under research [56], e.g., a food recommendation with a description of nutritional values may be hard for patients with low medical literacy. Therefore, introducing a personalized real-time recommender system that provides diet plans and physical exercise charts for all categories of patients with the disease will be of great use. The classification of results is based on the severity/inclination towards T2DM. The predictive classes are diabetic, low-risk, moderate-risk, and high-risk non-diabetic patients, as shown in Figure 18. Based on the prediction results, users can obtain the required diet plans and physical exercise charts.

### 4.4. Case Example

The recommender system generates personalized lifestyle guidance for each risk category identified by the ANN and fuzzy logic model. Based on a user’s symptoms, demographic profile, and lifestyle patterns, the system provides tailored diet plans, physical activity recommendations, behavioral modifications, and monitoring strategies as presented in Table 10. These individualized recommendations aim to support preventive, corrective, and therapeutic interventions according to the severity of diabetes risk. The integration of a personalized recommender system bridges the gap between risk prediction and actionable lifestyle management. By offering class-specific, affordable, and practical recommendations, the framework enhances user engagement and supports early intervention, particularly for individuals in high-risk or pre-diabetic states. This ensures that the model is not merely predictive but also clinically meaningful, promoting real-world impact in diabetes prevention and management.

## 5. Conclusions and Future Scope

This study presents a comprehensive AI-driven framework for the early prediction and personalized management of Type 2 Diabetes Mellitus (T2DM) using real-world lifestyle indicators. The proposed model combines an Artificial Neural Network with fuzzy logic to generate accurate multi-class risk predictions while simultaneously delivering individualized lifestyle, diet, and physical activity recommendations through an integrated recommender system. The framework has been developed, validated, and assessed with support from domain experts, including endocrinologists, diabetologists, and nutritionists, ensuring clinical relevance and practical usability. Implemented as a user-friendly web application, the system provides cost-effective and accessible support for individuals at varying levels of risk, enabling informed decision-making and encouraging sustainable behavioral modifications. By incorporating web-scraped food pricing data and expert-curated menus, the recommender engine offers economically feasible diet options, enhancing accessibility for diverse socioeconomic groups. Overall, the study demonstrates that lifestyle-centric AI models can complement standard diagnostic pathways and contribute meaningfully to preventive care and early intervention strategies for T2DM.

Although the framework demonstrates strong predictive capability and applicability, several limitations remain. First, the dataset is derived from a specific regional population, which may limit generalizability across broader demographic, cultural, or dietary contexts. Second, the model relies on lifestyle parameters and does not incorporate clinical biomarkers, laboratory measurements, or genetic predispositions, which could enhance diagnostic precision. Third, the recommender system provides structured lifestyle and dietary suggestions but does not account for personalized factors such as food allergies, comorbidities, financial constraints, or preferences beyond cost efficiency. Additionally, the model does not learn from user feedback or longitudinal changes, and its performance depends on static inputs rather than dynamic monitoring.

Future work can expand the system into a more robust and adaptive decision support framework. Integration with wearable devices, such as glucometers, smartwatches, and fitness trackers, will enable real-time monitoring of physiological and behavioral parameters, supporting continuous risk assessment. Incorporating clinical biomarkers, genetic indicators, and medical history can improve predictive accuracy and support comprehensive T2DM management. The recommender system may be enhanced using reinforcement learning or context-aware engines that adapt to user feedback, preferences, and behavioral patterns over time. Scaling the framework to diverse geographical regions and cultural dietary practices will improve generalizability. Finally, adopting distributed health data architectures or federated learning approaches will allow secure analysis of large-scale heterogeneous datasets, advancing proactive and personalized T2DM prevention strategies.

## Figures and Tables

**Table 1:** Attribute information of the dataset.

S. No	Parameter	Description	Measurement	Value Range
1	Age	Age of the participant in years.	Numeric	5 to 83
2	Sex	Gender of the participant.	Categorical	0 or 1
3	Family History	The presence or history of diabetes among any family members of the participant.	Categorical	0 or 1
4	Smoking	If the participant is smoker.	Categorical	0 or 1
5	Drinking	If the participant is liquor.	Categorical	0 or 1
6	Thirst	Number of times a participant drinks water in a day/night.	Numeric	1 to 15
7	Urination	Number of times the participant passes urine in a day/night.	Numeric	2 to 15
8	Height	Height of the participant in centimeters (cm).	Numeric	61 to 766
9	Weight	Weight of the participant in kilograms (Kg).	Numeric	15 to 96
10	Fatigue	If the participant feels fatigued.	Categorical	0 or 1
11	Outcome	If the participant has diabetes.	Categorical	0 or 1

**Table 2:** Descriptive statistics of the diabetes mellitus dataset.

Attribute	Count	Mean	Std	Min	25%	50%	75%	Max
Age	1939	41.77	15.84	5	31	39	50	83
Sex		0.47	0.50	0	0	0	1	1
Family History		0.27	0.45	0	0	0	1	1
Smoking		0.15	0.35	0	0	0	0	1
Drinking		0.18	0.38	0	0	0	0	1
Thirst		6.18	2.43	1	5	6	7	15
Urination		6.40	3.46	2	3	5	10	15
Height		161.60	33.14	61	154	162	167	195
Weight		61.65	11.48	15	55	62	69	96
Fatigue		0.69	0.46	0	0	1	1	1
Outcome		050	0.50	0	0	1	1	1

**Table 3:** Age-cohort distribution of participants and corresponding prevalence of Type 2 Diabetes.

Age Group	No. of Participants	% of Total	T2DM Cases	Prevalence (%)
5–17	112	5.7%	3	2.7%
18–30	384	19.8%	41	10.7%
31–45	612	31.6%	148	24.2%
46–60	517	26.7%	201	38.8%
>60	314	16.2%	149	47.5%

**Table 4:** Cramer’s V coefficients and *p*-values for lifestyle factors with respect to T2DM outcome.

Feature vs. Outcome	Cramer’s V	*p*-Value	Significance
Age	0.52	<0.001	Highly Significant (older age ↑ risk)
Sex	0.035	0.35	Not Significant (no clear sex difference)
Family History	0.053	<0.001	Highly Significant (family history ↑ risk)
Smoking	0.11	0.20	Not Significant (negligible association)
Drinking	0.19	0.02	Statistically Significant (slight association)
Thirst	0.43	<0.001	Highly Significant (frequent thirst ↑ in diabetics)
Urination	0.79	<0.001	Highly Significant (frequent urination ↑ in diabetics)
Height	0.21	0.01	Statistically Significant (slight inverse correlation)
Weight	0.33	<0.001	Highly Significant (higher weight ↑ risk)
Fatigue	0.64	<0.001	Highly Significant (fatigue more common in T2DM)

Cramer’s V is a non-directional measure of association ranging from 0 to 1; therefore, it does not encode the sign of a relationship. The upward (↑) in the “Significance” column indicate the observed trend in the dataset, i.e., whether higher values of the feature occur more frequently among T2DM cases based on groupwise comparisons of feature distributions. These arrows represent descriptive trends rather than the statistical “direction” of correlation.

**Table 5:** ANN architecture, training, and testing configurations.

Parameter Category	Description/Value
Dataset Split	70% Training, 30% Testing
Input Features	Lifestyle indicators: age, thirst, urination, fatigue, weight, height, etc.
Number of Classes	4 (A: Low-risk, B: Moderate-risk, C: High-risk, D: T2DM)
ANN Architecture	Input layer → 3 Hidden Layers → Output Layer
Hidden Layer Sizes	Layer 1: 64 neurons. Layer 2: 32 neurons. Layer 3: 16 neurons
Activation Functions	ReLU (Hidden Layers), Softmax (Output Layer)
Output Representation	One-hot encoding for A, B, C, and D
Loss Function	Categorical Cross-Entropy
Optimizer	Adam
Learning Rate	0.001
Batch Size	32
Epochs	150 (with Early Stopping)
Weight Initialization	Xavier (Glorot Uniform)
Regularization Techniques	Dropout (0.2), L2 (0.001)
Performance Metrics	Accuracy, Precision, Recall, F1-score, ROC–AUC
Hardware Used	Intel i5 Processor, 16 GB RAM, NVIDIA, 256 SSD, and 1 TB HDD
Software/Environment	Python 3.x, TensorFlow/Keras, NumPy, Pandas

**Table 7:** Comparative analysis of our proposed model with state-of-the-art methods.

Author	Model	Accuracy	Precision	Recall	F1-Score	AUC-ROC
Singh and Singh [48]	NSGA-II, RBF, linear SVM, polynomial SVM, DT, KNN	91.90 with NSGA-II	93.30	99.30	96.20	92.00
Wang et al. [49]	DT, RF, GB, XGB, LGBM, MLP, ensemble LightGBM-XGB-GB,	77.92 with ensemble model	75.70	75.00	75.34	77.65
Liu et al. [50]	LR, RF, XGB, ensemble model	66.60 with ensemble model	-	-	-	-
Yang et al. [51]	LDA, RF, SVM, ensemble model	73.00 with ensemble model	38.90	81.90	52.75	84.90
Sivashankari et al. [52]	RF, KNN, DT, GB, and NB, LR, ensemble model	93.10 with ensemble model	84.00	83.90	83.50	90.00
Our Paper	Fuzzy-ANN Model	93.64	94.00	93.50	93.50	94.07

NSGA-II: Non-Dominated Sorting Genetic Algorithm II, RBF: Radial Basis Function, SVM: Support Vector Machine, DT: Decision Tree, RF: Random Forest, GB: Gradient Boosting, XGB: Extreme Gradient Boosting, LGBM: Light Gradient Boosting Machine, LDA: Linear Discriminant Analysis, KNN: K-Nearest Neighbors, NB: Naive Bayes, LR: Logistic Regression, MLP: Multilayer Perceptron, and ANN: Artificial Neural Network.

**Table 8:** Exercise chart for diabetic patients.

Exercise Chart 1—Diabetic Patients	
**WALKING**	It will reduce the complications of blood sugar levels and support weight loss in people with diabetes.
**CYCLING**	When individuals experience lower joint pain due to diabetes neuropathy, a condition involving nerve damage, cycling can serve as an effective means to achieve fitness objectives while minimizing strain on the joints.
**SWIMMING**	Engaging in aquatic activities offers an additional joint-friendly exercise option. Activities such as swimming, water aerobics, aqua jogging, and other water-based exercises can provide a comprehensive workout for the heart, lungs, and muscles, all while imposing minimal stress on the joints.
**TEAM SPORTS**	Numerous recreational sports can provide excellent aerobic workouts. Consider exploring options like basketball, soccer, softball, doubles tennis, and ultimate frisbee.
**YOGA**	Regular physical activity can significantly benefit individuals living with Type 2 Diabetes Mellitus by assisting in the management of blood sugar, cholesterol levels, and weight. Additionally, it has the potential to reduce blood pressure, enhance the quality of sleep, and uplift one’s mood.

**Table 9:** Exercise chart for non-diabetic patients.

Exercise Chart 2—Non-Diabetic Patients	
**WALKING**	It will reduce the complications of blood sugar levels and support weight loss in people having diabetes.
**CYCLING**	When individuals experience lower joint pain due to diabetes neuropathy, a condition involving nerve damage, cycling can serve as an effective means to achieve fitness objectives while minimizing strain on the joints.
**SWIMMING**	Engaging in aquatic activities offers an additional joint-friendly exercise option. Activities such as swimming, water aerobics, aqua jogging, and other water-based exercises can provide a comprehensive workout for the heart, lungs, and muscles, all while imposing minimal stress on the joints.
**TEAM SPORTS**	Numerous recreational sports can provide excellent aerobic workouts. Consider exploring options like basketball, soccer, softball, doubles tennis, and ultimate frisbee.
**YOGA**	Regular physical activity can significantly benefit individuals living with Type 2 Diabetes Mellitus by assisting in the management of blood sugar, cholesterol levels, and weight. Additionally, it has the potential to reduce blood pressure, enhance the quality of sleep, and uplift one’s mood.
**JOGGING**	Jogging can be a form of exercise for people who have inclination towards diabetes as it helps improve the body’s sensitivity to insulin.
**OTHER EXERCISES**	Pushups. Squats. Dumbbell Rows. Side Planks. Gym Weights-Based Exercise.

**Table 10:** Personalized lifestyle recommendations generated by the proposed recommender system for each diabetes risk class (A–D).

Class	Profile	Recommendation
Low-Risk Individual (Class A)	Age 29 Normal BMI No family history Mild occasional thirst Generally active lifestyle	Maintain current physical activity (25–30 min brisk walking/day). Adopt balanced meals with 50–55% complex carbohydrates, lean proteins, and low saturated fat. Limit sugary beverages and late-night snacking.
Moderate-Risk Individual (Class B)	Age 32 Mild thirst Active lifestyle	Maintain diet plan 2 Reduce fried food 25–30 min walking
High-Risk Individual (Class C)	Age 55 Persistent fatigue, thirst = 10/day Weight 84 kg Family history Yes	1500–1700 kcal/day economical diet plan Morning walking: 40 min/day Fresh vegetables from BigBasket (low-cost items list) Replace sugar with stevia Evening cycling or swimming recommended
Diabetic (Class D)	Age 62 Individual with very high thirst Constant fatigue Rapid weight fluctuation Symptomatic hyperglycemia.	Doctor consultation Detailed diabetic diet chart + structured exercise routine + glycemic index-based food selection.

## Data Availability

The raw data supporting the conclusions of this article will be made available by the authors on request.
